# A DNA-Based Assay for Digoxin Detection

**DOI:** 10.3390/bios8010019

**Published:** 2018-03-06

**Authors:** Michael V. Kjelstrup, Line D. F. Nielsen, Malthe Hansen-Bruhn, Kurt V. Gothelf

**Affiliations:** Interdisciplinary Nanoscience Center (iNANO) and Department of Chemistry, Aarhus University, Gustav Wieds Vej 14, 8000 Aarhus C, Denmark; Michael.Vingborg.Kjelstrup@zacco.com (M.V.K); debois@inano.au.dk (L.D.F.N); mhb@inano.au.dk (M.H.-B.)

**Keywords:** digoxin detection, homogeneous assay, DNA nanotechnology, DNA strand displacement competition reaction, detection of small molecules, detection in plasma

## Abstract

The most common method for quantifying small-molecule drugs in blood samples is by liquid chromatography in combination with mass spectrometry. Few immuno-based assays are available for the detection of small-molecule drugs in blood. Here we report on a homogeneous assay that enables detection of the concentration of digoxin spiked into in a plasma sample. The assay is based on a shift in the equilibrium of a DNA strand displacement competition reaction, and can be performed in 30 min for concentrations above 10 nM. The equilibrium shift occurs upon binding of anti-digoxigenin antibody. As a model, the assay provides a potential alternative to current small-molecule detection methods used for therapeutic drug monitoring.

## 1. Introduction

In clinical laboratories, small-molecule drugs in blood samples are commonly quantified using liquid chromatography in combination with mass spectrometry [1,2,3]. However, these methods often require hours to provide the blood concentration of the drug. Alternatively, binding assays such as ELISA (enzyme-linked immunosorbent assay) may be used for the detection of analytes. These methods rely on immobilizations of the binding moiety to a surface, and may require elaborate technical procedures or lack precision in the quantification of small-molecule drugs [4]. Another choice is a homogeneous binding assay. The advantage of homogeneous binding assays is that they can be performed in a single tube containing the specimen and all other reagents. No immobilization, separation, or washing steps are required [5]. Many homogenous assays are based on either aptamers [6,7] or DNA-binding proteins [8,9]. Other assays could be quenchbodies [10,11], binding-induced annealing [12,13,14,15,16], or proximity ligation [17,18,19]. The latter assays often involve multivalent binding or DNA–protein conjugation. Additionally, economic pressure in the health-care system has pushed the focus more towards less time- and cost-consuming solutions [20].

Here we report on a homogeneous assay that requires neither immobilization, multivalent binding, nor DNA-protein conjugation. It provides an assessment of the concentration of digoxin in a sample of unknown concentration. Digoxin is used in the treatment of various heart diseases, such as atrial fibrillation, atrial flutter, and even heart failure [21].

**Figure 1:**
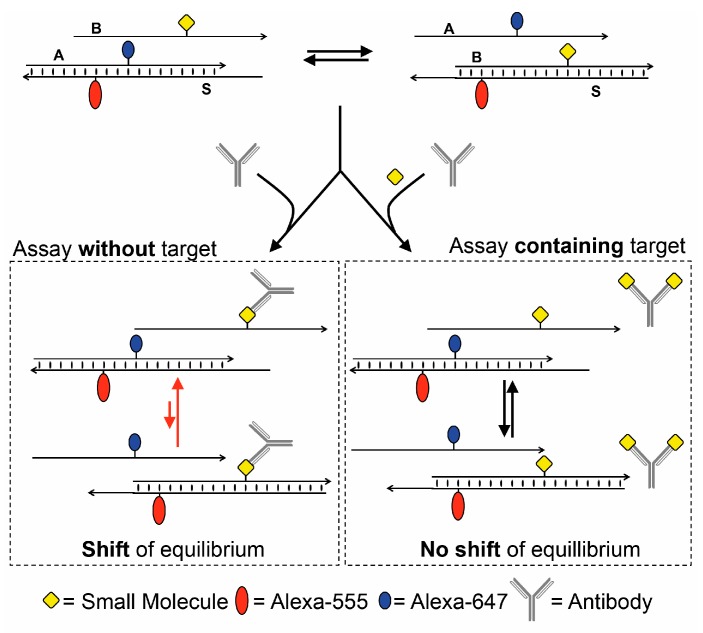
Overview of the strand displacement competition assay. DNA strands A (conjugated to Alexa-647), B (conjugated to digoxigenin), and S (conjugated to Alexa-555). The two toeholds on S are complimentary to sequences on A and B, respectively. The constant region on S is complimentary to both A and B. In the absence of an anti-digoxigenin antibody, the system obtains a low Förster resonance energy transfer (FRET) state, and a high FRET state in the presence of an antibody. If an antibody binds to free digoxigenin or digoxin, it is blocked and will not influence the equilibrium.

Based on the previously reported strand displacement competition (SDC) assay [22], we developed a generic assay for the detection of small-molecule analytes [23]. In brief, this assay is constituted of three DNA strands A, B, and S, where A and B compete for the binding to S by toehold-mediated strand displacement (Figure 1).

The state of the equilibrium is monitored by Förster resonance energy transfer (FRET) [24,25] by dyes conjugated to the A and S strands. For application of this assay to the detection of proteins and in turn small molecules, the small-molecule target is conjugated to a base in the B strand [26,27]. The equilibrium depends on the melting temperature of the AS and BS duplexes. The small molecule conjugated to the B strand has a minor impact on the melting temperature. The system is designed to thermodynamically favor the BS duplex, hence providing a low FRET state, since the dyes located on the S and A strand are separated. 

Upon addition of a target protein that binds to the small molecule on B, the melting temperature of the BS duplex decreases [23], causing a shift of the equilibrium towards the AS duplex, which results in a high FRET state. When adding a solution of the free small molecule to the ABS and protein mixture, it may outcompete the binding to the B strand, thereby shifting the equilibrium back to the initial FRET state (Figure 1). This system allows the detection of both the protein and the small molecule through the FRET readout.

In our previous report, we described the detection of digoxigenin as a model compound by the SDC method using anti-digoxigenin antibody (aD) [23]. In the previous assay, 24 h incubation was required for the detection of digoxigenin. Here, we present a thorough investigation and optimization of the assay for 30-min detection of digoxin for concentrations above 10 nM. The assay was also optimized for detection in the very low therapeutic range of digoxin in blood of 1.2–2 nM, but with longer assay times.

## 2. Materials and Methods

### 2.1. General

All used reagents are commercially available. The reagents were purchased at the highest possible quality from Sigma-Aldrich, Link Technologies, Thermo and Berry & Associates, and used without further purification.

All DNA oligonucleotides were synthesized with an in-house BioAutomation MerMade-12 oligonucleotide synthesizer, using standard or modified phosphoramidites according to instructions, and afterwards precipitated by ethanol precipitation. All synthesized DNA strands were used in conjugation reactions without further purification. The water used for the DNA experiments was purified on a MilliQ system. Reverse-phase HPLC was performed on a Hewlett Packard Agilent 1100 Series using Phenomenex Clarity 3u Oligo-RP 50 × 4.6 mm columns. All concentrations of oligonucleotides were determined using a Thermo Fisher Scientific ND-1000 NanoDrop spectrophotometer. Anti-digoxigenin antibody was acquired from Roche Diagnostics via Sigma-Aldrich. All concentrations of antibodies were calculated based on a molecular weight of 150.000 Da (IgG). All NHS (N-hydroxysuccinimide) esters (Digoxigenin-NHS ester, Alexa647-NHS ester and Alexa555-NHS ester) were purchased as powders and dissolved in DMSO and aliquoted (100 µg in each), freeze-dried, and stored at −20 °C.

### 2.2. Construction of the Assay for Overnight Detection

BILATEC tubes (0.5 mL, Sigma-Aldrich, St. Louis, MO, USA) were used for all the reactions. An assay was prepared by mixing DNA strands A, B, and S in equal stoichiometric ratio (e.g., 70 μL, 20 nM), in 1 × TAE-Mg buffer (tris base, 40 mM; acetic acid, 20 mM; EDTA, 2 mM; magnesium, 12.5 mM) at pH 8. The anti-digoxigenin antibody and H_2_O/digoxin was also added at the same time. Then, the whole mixture was incubated at room temperature overnight in the dark. Since this method is based on the thermodynamic equilibrium, the order of addition of different components is irrelevant to the final readout.

### 2.3. Construction of the Assay for 30-Minute Detection

The sample with or without digoxin was first premixed with the anti-digoxigenin antibody. After incubation at room temperature for the selected time (e.g., 10 min), the mixture was added into the standard assay (the A-, B-, and S-strand) and incubated at room temperature for another selected timeframe (e.g., 20 min). For plasma experiments, water was substituted with EDTA-buffered plasma (i.e., A, B, S, anti-digoxigenin antibody, digoxin, buffer, EDTA-buffered plasma to 70 μL).

### 2.4. FRET Experiments

Fluorescence measurements were performed using a scanning spectrofluorometer (Fluoro-Max-3 or Fluoro-Max-4, HORIBA Jobin Yvon Inc., Upsala, Sweden). The assay-specimen mixture was pipetted into a quartz cuvette (70 μL), which was washed by 1 × TAE-Mg three times between different samples. All spectral measurements were recorded with 0.5–2 s integration time and 1 nm wavelength interval at 25 °C. For the FRET pair of Alexa-555 and Alexa-647, excitation was performed at 550 nm for Alexa-555. Relative FRET efficiencies were calculated as Er = IDA/(IDA + IDD), where IDA is the acceptor peak fluorescence intensity at 667 nm and IDD is the donor peak fluorescence intensity at 567 nm.

### 2.5. Blood Samples

EDTA-buffered human blood was acquired from the local blood bank (Aarhus University Hospital, Denmark) from an unknown healthy person. The blood was centrifuged at 3.000 rcf (relative centrifugal force) for 15 min at room temperature to separate the cells from the plasma, after which the plasma was spiked.

### 2.6. DNA Sequences

The core sequences in this study were from Zhang and Winfree’s previous study, which were designed to exhibit minimal secondary structure and crosstalk [28]. See Tables S1 and S2 for sequences and structures, respectively. 

### 2.7. DNA-Ligand/Fluorophore Conjugation by NHS Ester-Amine Reaction

Amine-modified DNA (25 μL, 100 μM) was mixed with NHS ester (100 μg) dissolved in DMSO or DMF (50 μL). The mixture was added to 20 mM sodium carbonate buffer (20 mM, pH 8.5; 25 μL) and left reacting at 25 °C for 1 to 2 h (alternatively, overnight). The mixture was purified by ethanol precipitation followed by HPLC purification; and its concentration was determined by UV absorption at 260 nm.

### 2.8. Calculation of Limit of Detection (LOD)

Limits of detection were calculated using Equation (1) [29]. Here, ΔFRET changes are the slopes (FRET ratio over digoxin concentration) of the linear parts of the 10 nM concentration study with 30-min detection (Figure 8 and Figure S3) and the 2 nM concentration study with overnight detection (Figure 10 and Figure S4). The standard deviations, σ, are the standard deviations of the lowest concentration from the concentration curves (standard deviation at 10 nM and 1 nM, respectively).

(1) $$\mathrm{LOD}=\frac{3*\sigma \left(\mathrm{low}\;\mathrm{concentration}\right)}{\Delta \mathrm{FRET}\;\mathrm{change}}$$

## 3. Results and Discussion

**Figure 2:**
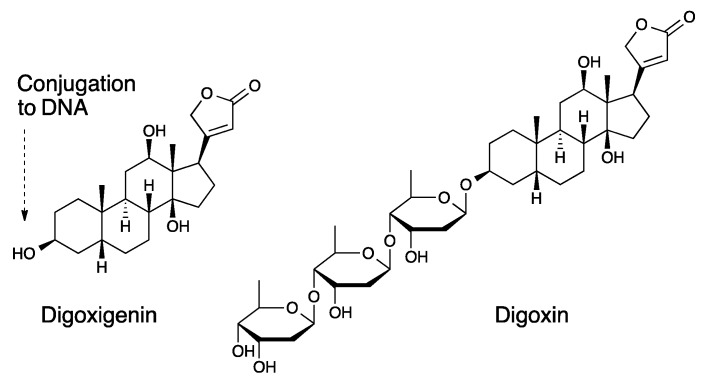
Digoxigenin and digoxin structure comparison. The OH group on digoxigenin is used for conjugation to the DNA B strand.

The structure of digoxigenin and the steroid fraction of digoxin are identical; however, digoxin additionally contains a trisaccharide chain (Figure 2). In the digoxigenin assay, digoxigenin is linked to the DNA chain B via the hydroxyl group indicated by an arrow in Figure 2.

**Figure 3:**
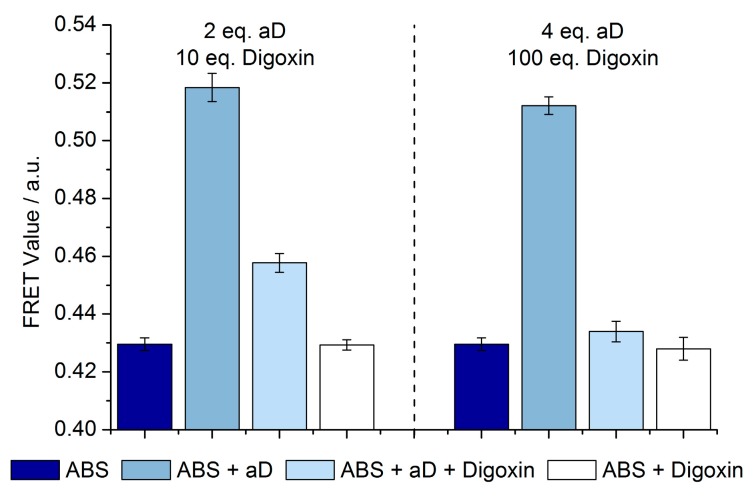
Addition of free digoxin to the digoxigenin assays. The FRET values for overnight analysis of anti-digoxigenin antibody (aD) and digoxin with the strand displacement competition (SDC) assay (20 nM, A:B:S = 1:1:1). The assay shifted from lower FRET to higher FRET when aD (40 or 80 nM, 2 or 4 eq. (equivalents)) was present, and it shifted back when digoxin (200 or 2000 nM, 10 or 100 eq.) was present as well. Three samples for each measurement (six for ABS). Plotted values are mean values with standard deviations compared to the mean value. All samples were incubated overnight before measuring.

Since aD binds to digoxigenin even when linked to a DNA strand, and the specificity for the commercial antibody is towards both digoxigenin and digoxin (96% cross-reactivity) [30], we hypothesize that the assay used for digoxigenin could be extended to detecting digoxin (Figure 3). When adding aD to a solution of A-, B- (conjugated to digoxigenin), and S-strand, a shift of the equilibrium towards the AS duplex occurs. This was observed as an increase in the FRET value (Figure 3, columns 1 versus 2 and 5 versus 6). A shift back to the low FRET state was observed after adding free digoxin to the SDC assay solution while incubating overnight (Figure 3, column 2 versus 3 and 6 versus 7). This experiment verifies that it is possible to exchange free digoxigenin molecules for free digoxin molecules without compromising the shift between the FRET states in the original assay.

**Figure 4:**
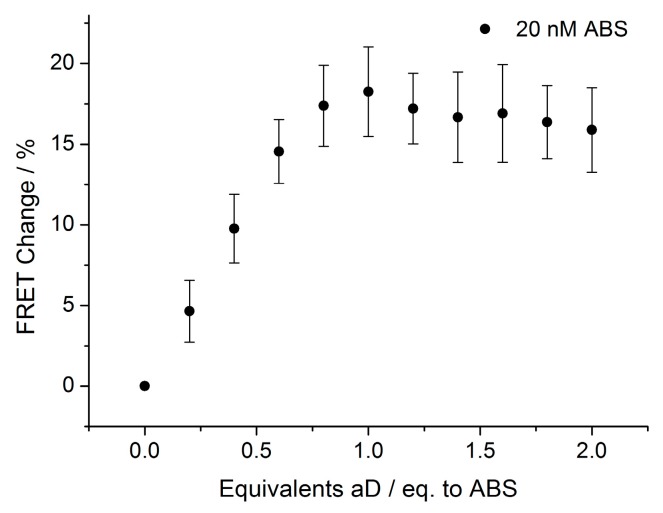
aD titration after 20 min of incubation. FRET change for the titration of the digoxigenin SDC assay (20 nM, A:B:S = 1:1:1) with aD. Experiment was performed in triplicate. The plotted values are mean values with standard deviations compared to the mean value. ABS and aD were incubated for 20 min before measuring the sample. Zero equivalents are defined as zero, and the FRET change is calculated on this basis.

When analyzing the optimal ratio between antibody and DNA strands (Figure 4), a linear correlation was observed between zero and one equivalent of antibody to DNA strands, while the addition beyond one equivalent resulted in no significant additional change.

Since the antibody has two binding sites, we anticipated that only 0.5 equivalents of aD should be required to obtain saturation; however, this is believed to reflect the actual activity of the commercially available aD antibody.

**Figure 5:**
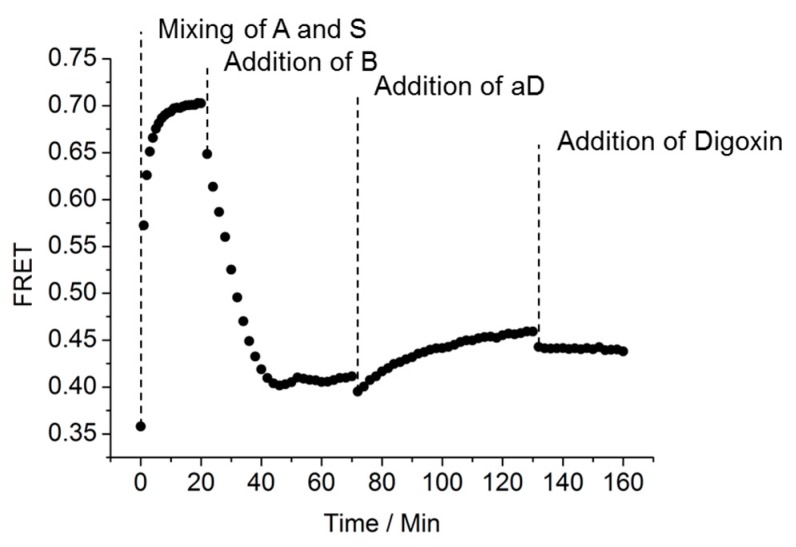
Real-time study of the change in FRET value over time when mixing SDC assay: Mixing of A and S (20 nM, A:S = 1:1), addition of B (20 nM, 1 eq.) to the AS solution, addition of aD (20 nM, 1 eq.) to the ABS solution, addition of digoxin (200 nM, 10 eq.) to the ABS-aD solution. Zero (0) min is defined as data obtained right after mixing of A and S.

One of the drawbacks of the previously-reported SDC assay was the duration of the incubation time. To optimize the speed of the digoxin assay, we first undertook an analysis of the real-time behavior. The FRET responses to adding the different constituents of the assay over time are shown in Figure 5. Upon mixing the A- and S-strands, an increase in FRET value was observed. Upon addition of the B-strand, the equilibrium started to settle over the next 20–30 min. This was observed as a decrease of the FRET value.

After adding aD, a slow increase in FRET value transpired (i.e., the equilibrium shifted towards the AS duplex) over the next 60 min. However, after addition of digoxin to the solution the FRET value did not increase, as would have been expected, over the next 30 min. This lack of increase in FRET value indicates a slow off-rate for the antibody. The slight decrease in FRET which was observed after each addition is probably a dilution effect. The mixing of ABS and aD followed by incubating for 24 h, before the addition of an excess of digoxigenin did not lead to any significant change of the FRET value after 60 min either (data not shown). These results further underline that the exchange of the aD binding is slow.

**Figure 6:**
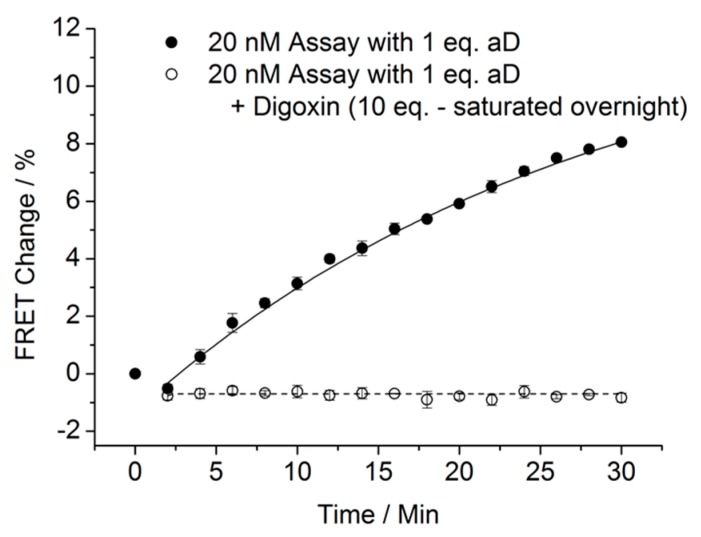
Inhibition of the aD with digoxin before addition to the SDC assay (20 nM, A:B:S = 1:1:1) and measurement of the FRET change over 30 min when adding either aD (20 nM, 1 eq.) (black) or adding aD (20 nM, 1 eq.) after overnight saturation with digoxin (200 nM, 10 eq.) (light gray). Experiments were performed in triplicate. The plotted values are mean values with standard deviations compared to the mean value. The FRET value at zero min (only ABS) is defined as zero, and FRET change is calculated on this basis.

To avoid the very slow exchange of the antibody between binding to the DNA-linked digoxigenin and digoxin in solution, we instead investigated premixing aD with digoxin overnight before adding it to the ABS mixture followed by measuring the change in FRET values (Figure 6). Thereby, we observed a large difference in FRET values when comparing the incubation with aD alone versus the mixture of aD and digoxin. We define the premixing of the antibody and the ligand sample before addition to the ABS as the “inhibition” method.

**Figure 7:**
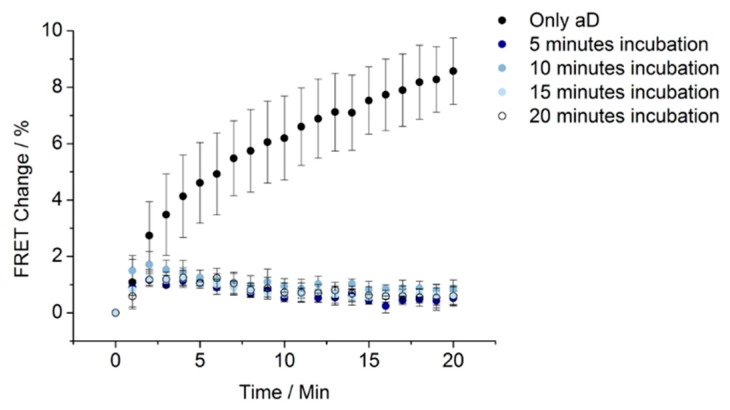
Influence of the incubation time of aD with digoxin for the saturation of aD. The FRET change was monitored over 20 min after adding aD (20 nM, 1 eq.) or adding aD (20 nM, 1 eq.) after saturation of aD with digoxin (200 nM, 10 eq.) for different periods of time. Experiments were performed in triplicate. The plotted values are mean values with standard deviations compared to the mean value. The FRET value at zero (0) min (only ABS) is defined as zero (0), and FRET change is calculated on this basis.

In order to reduce the total assay time, we investigated if the incubation time between aD and digoxin could be decreased while maintaining a significant shift in FRET value between the non-saturated and the saturated assay (saturated with an excess of 10 equivalents of digoxin). Five assays were constructed, where the incubation time was varied from 5 to 20 min for the saturation. The data are depicted in Figure 7. It is observed that aD was saturated at the applied saturation times, and only an absence of digoxin caused aD to change FRET value. Hence, the saturation time for aD with digoxin is fast (<5 min).

**Figure 8:**
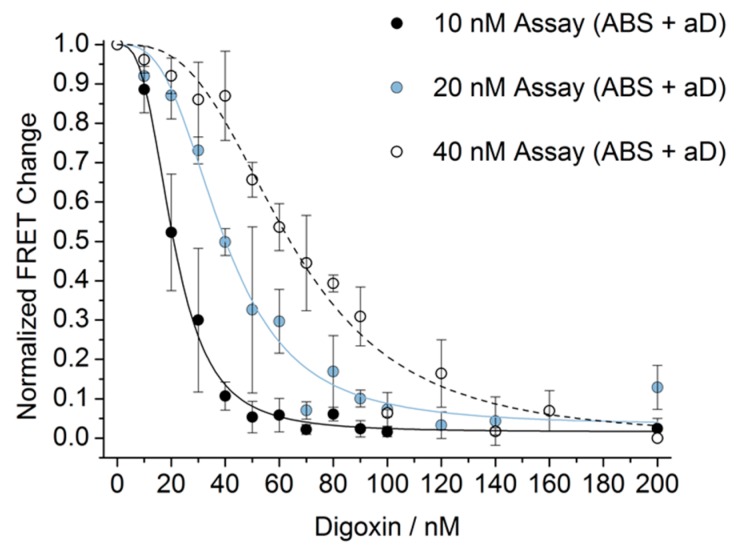
A three-assay setup. Normalized FRET change for three different SDC assay concentrations (A:B:S:aD = 1:1:1:1) relative to variable digoxin concentrations (0 to 200 nM). aD was incubated for 10 min with the digoxin sample of the given concentration and was then added to ABS followed by 20 min incubation (total incubation/assay time of 30 min). Experiments were performed in triplicate. The plotted values are mean values with standard deviations compared to the mean value.

Next, we used the assay to measure various concentrations of digoxin (0 to 200 nM) (Figure 8). The saturation point of the aD was highly dependent on the concentration of the assay, and in particular the concentration of aD in the assay. Therefore, we monitored the response to digoxin in assays of three different concentrations: 10 nM, 20 nM, and 40 nM (A-, B-, and S-strand, and aD, 1:1:1:1) as shown in Figure 8.

By applying multiple SDC assays of different concentrations, samples containing digoxin of unknown concentration can be determined within 30 min of analysis time. Utilizing the application of multiple assays around the concentration range of interest, an estimate of the digoxin concentration in an unknown sample can be obtained. If more assays with a shift in the desired concentration range are applied, a more precise concentration assessment will be obtained. The detection limit for the three-assay setup shown was 10 nM of digoxin, as this is the concentration where a slight shift in the equilibrium of one of the assays was obtained. The concentration range for the three-assay setup shown in Figure 8 is from 10 nM to 100 nM digoxin. At concentrations above 100 nM, all three assays were almost completely saturated. It is worth noting that the uncertainties for a specific concentration of digoxin were not overlapping between any of the three assays when analyzing the concentration range. It is also worth noting that the results indicate that four times the concentration of digoxin compared to the assay is needed to fully saturate the assay.

**Figure 9:**
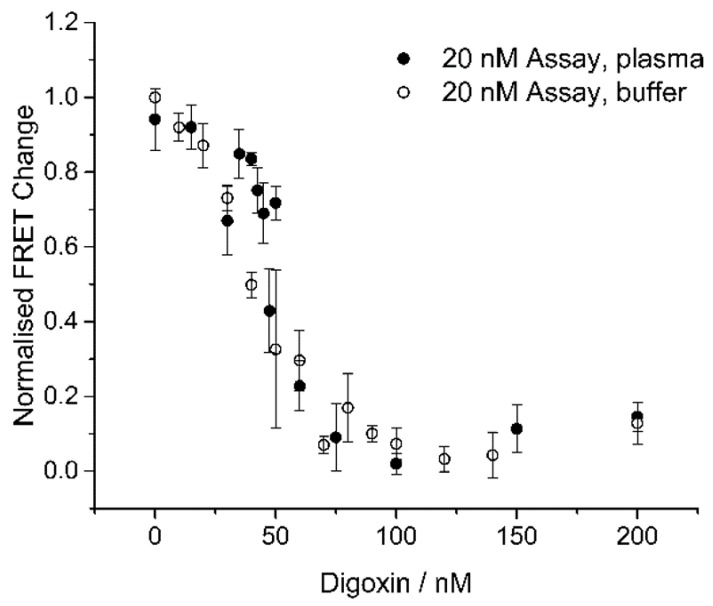
Comparison of 20 nM assay in buffer and in plasma. Normalized FRET change (A:B:S:aD = 1:1:1:1) relative to variable digoxin concentrations (0 to 200 nM) in buffer and in plasma (56–61%). aD was incubated for 10 min with the digoxin sample of the given concentration, and was then added to ABS followed by 20 min incubation (total incubation/assay time of 30 min). Experiments were performed in triplicate. The plotted values are mean values with standard deviations compared to the mean value.

Finally, we tested the assay for the detection of digoxin in human plasma spiked with digoxin, and we compared the results to the detection in a buffered system (Figure 9). The human blood samples were centrifuged to remove red- and white blood cells, after which the plasma was spiked with digoxin. As revealed in Figure 9, the assay had similar sensitivity to digoxin in plasma (≥56%) and in buffer. One of the inherent advantages of the SDC assay is that changes in the environment such as buffer, temperature, etc. had similar effects on both sides of the equilibrium, and hence the assay is robust towards detection in complex matrices under various conditions. Detection in plasma can often be a problem in FRET-based systems due to the autofluorescence arising from biomolecules, but this problem was not observed in our assay due to the excitation at a high wavelength (550 nm) (Figure S2).

**Figure 10:**
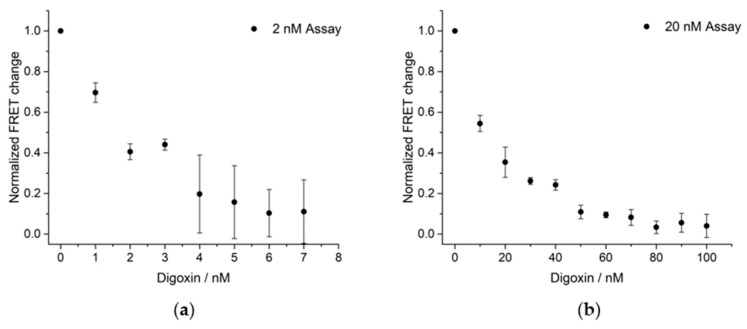
Detection of digoxin in the therapeutic range and comparison with 20 nM assay with overnight incubation. (**a**) A 2 nM assay for detection in the therapeutic range (1.2–2 nM). Normalized FRET change (A:B:S:aD = 1:1:1:1) relative to variable digoxin concentrations (0 to 7 nM) in buffer. All components were mixed at the same time and incubated overnight. Experiments were performed in triplicate. The plotted values are mean values with standard deviations compared to the mean value. (**b**) A 20 nM assay for comparison. Normalized FRET change (A:B:S:aD = 1:1:1:1) relative to variable digoxin concentrations (0 to 100 nM) in buffer. All components were mixed at the same time and incubated overnight. Experiments were performed in triplicate. The plotted values are mean values with standard deviations compared to the mean value.

It was attempted to detect digoxin at lower concentration to reach the relevant therapeutic range of digoxin in blood of 1.2–2 nM [31]. However, when decreasing the concentration of the assay, the sensitivity of the FRET readout in the 30 min assay did not allow precise detection below 10 nM. However, we were able to detect digoxin in the therapeutic range with an overnight readout. As shown in Figure 10a, the 2 nM assay exhibited a large shift in FRET between 1 nM and 2 nM of digoxin, which makes this assay suitable for detection of digoxin in the therapeutic range. The 2 nM assay was compared to a 20 nM assay with overnight detection (Figure 10b). Limits of detection (LODs) were calculated for both detection within 30 min and overnight detection (see Supplementary Materials for calculations). The LOD for 30 min detection was calculated to be 8.2 nM, while the LOD for overnight detection was calculated to be 1.08 nM. This correlates well with the findings that in the current 30 min detection setup we were not able to detect concentrations within the therapeutic index, but we were able to measure such low concentrations overnight. 

## 4. Conclusions

We constructed an SDC assay for the rapid detection of the pharmaceutically-relevant drug digoxin. Unlike the assays where the antibody or the analyte are immobilized, this solution-based SDC assay enables determination of digoxin when decreasing the concentration of the assay within a total timeframe of 30 min for concentrations above 10 nM. Most importantly, the assay also provided the efficient detection of digoxin in plasma. When the detection time was extended, it was also possible to detect digoxin in the therapeutic range with overnight detection. Efforts towards the rapid detection of digoxin within the therapeutic range within 30 min are currently being undertaken. The current assay is generic [23], and can be extended to the detection of other small molecules, and here digoxin has served as a model compound. By conjugation of other small-molecule analytes to the B strand shown in Figure 1, and application of an antibody or another protein that binds to the small-molecule analyte, it is very likely that the equilibrium in the SDC assay will be shifted. Hence, the assay has clear potential for the detection of other small-molecule analytes in an inhibition assay. Since most small-molecule drugs have higher therapeutic range than digoxin, the method may have potential for the clinical detection of other small-molecule drugs [32].

## Supplementary Materials

The following are available online at http://www.mdpi.com/2079-6374/8/1/19/s1. Figure S1: Emission spectra (corrected) of selected data points from experiment seen in Figure 7; Table S1: DNA sequences and mass spectrometry data; Table S2: Structure of modified bases and of the modified parts of the DNA strands after conjugation reactions; Figure S2: Emission spectrum of 57% plasma at excitation at 550 nm; Figure S3: FRET ratio as a function of the digoxin concentration in the linear range of experiment from Figure 8 (0–40 nM of digoxin); Figure S4: FRET ratio as a function of the digoxin concentration in the linear range of experiment from Figure 10 (0–4 nM of digoxin).
