# Supplementary material for: A DNA-Based Assay for Digoxin Detection
Source: Biosensors (Basel). 2018 Mar 6;8(1):19. doi: 10.3390/bios8010019 (PMC5872067; doi:10.3390/bios8010019)
Supplement: Supplementary file 1 [file biosensors-08-00019-s001.pdf]

Supplementary Materials: A Rapid DNA-Based Assay for Digoxin Detection

Michael V. Kjelstrup, Line D. F. Nielsen, Malthe Hansen-Bruhn and Kurt V. Gothelf

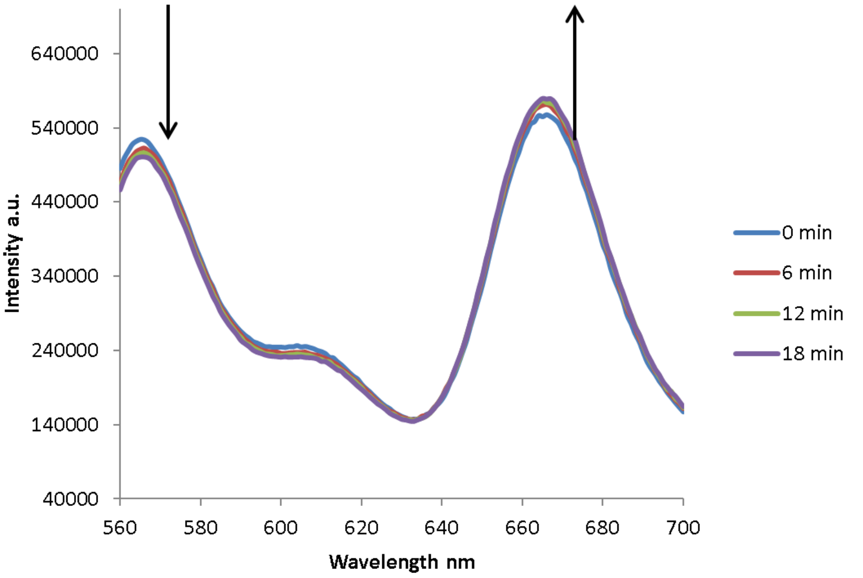

Figure S1: Emission spectra (corrected) of selected data points from experiment seen in Figure 7 (only aD) (λex = 550 nm). After addition of aD to the A, B, and S strand, the intensity of the fluorescent signal from Alexa-555 decreases, while the intensity of the fluorescent signal from Alexa-647 increases, hence an increase in FRET value is observed over time.

Table S1: DNA sequences and mass spectrometry data (Toehold regions are written in italic and written in color code (red/blue))

Name	Sequence (5′-)	Calculated Mass	Observed Mass
A4-NH	*CTCA*TTCAA(T-Amine1)ACCCTACG	5532,8 Da	5532,5 Da
A4-647	*CTCA*TTCAA(T-Alexa647)ACCCTACG	-*	6373.5 Da
B4_dU_NH	TTCAATACCC(dU-Amine2)ACG*TCTC*	5410.6 Da	5410.6 Da
B4_dU_D2	TTCAATACCC(dU-Dig)ACG*TCTC*	5954.3 Da	5953.7 Da
S66-NH	*TGGAGA*CG(T-Amine1)AGGGTATTGAA*TGAGGG*	8349.6 Da	8351.6 Da
S66-555	*TGGAGA*CG(T-Alexa555)AGGGTATTGAA*TGAGGG*	-*	9166.0 Da

* The exact masses of Alexa647 and Alexa555 are not publicly accessible.

(Two different amine-modified phosphoramidites have been used to synthesize the DNA strands. An Amino C6 dT (Amine1) was used for the synthesis of A4-NH and S66-NH, and an 5-Aminoallyl-dU (Amine2) was used for synthesis of B4_dU_NH)

Table S2: Structure of modified bases and of the modified parts of the DNA strands after conjugation reactions.

Name	Structure
Amino C6 dT (Amine1)	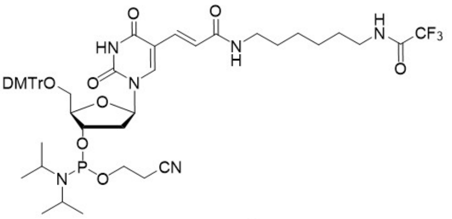
5-Aminoallyl-dU (Amine2)	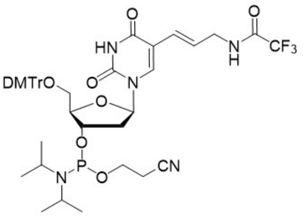
T-Alexa647	**No structure for Alexa-fluorophores**
dU-Dig	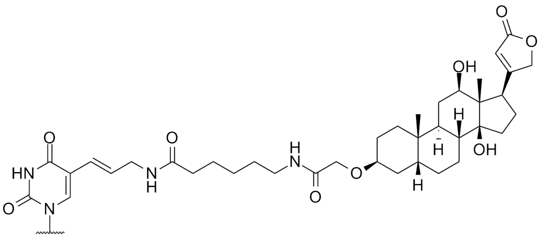
T-Alexa555	**No structure for Alexa-fluorophores**

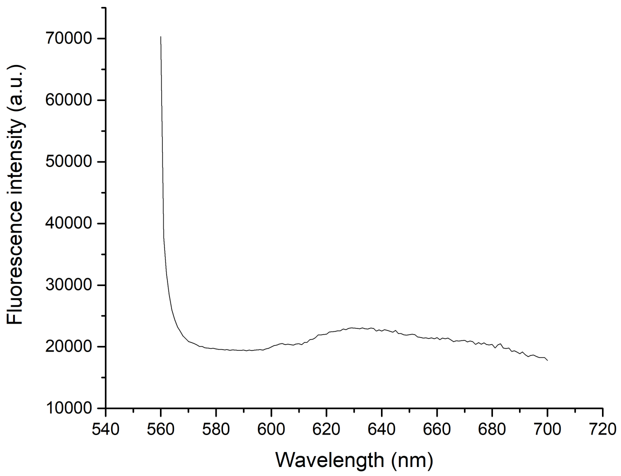

Firgure S2: Emission spectrum of 57 % plasma at excitation at 550 nm. The autofluorescence signal of 57 % plasma (plasma spiked with 1xTAE-Mg buffer) at excitation at 550 nm, is far less than the fluorescent signal from the assay (Figure S1), which makes it possible to use the assay in plasma.

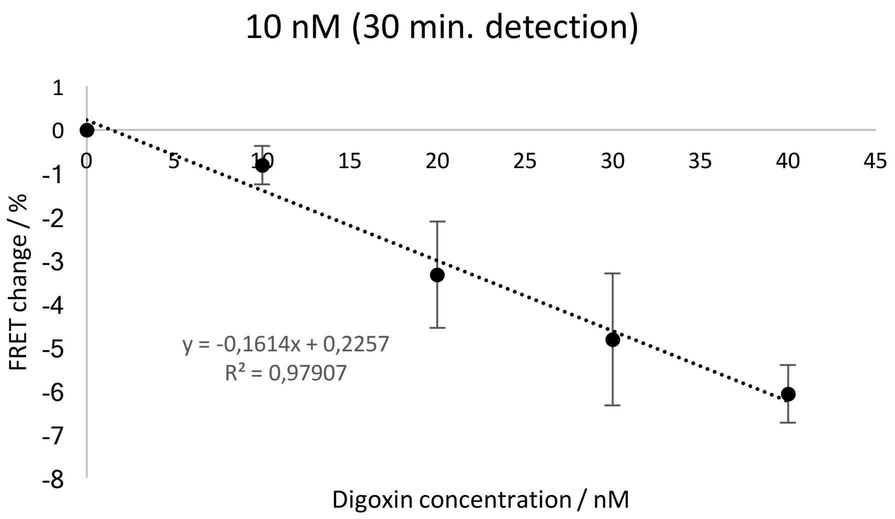

Figure S3: FRET ratio as a function of the digoxin concentration in the linear range of experiment from Figure 8 (0-40 nM of digoxin). LOD was calculated from the linear regression function (dashed line).

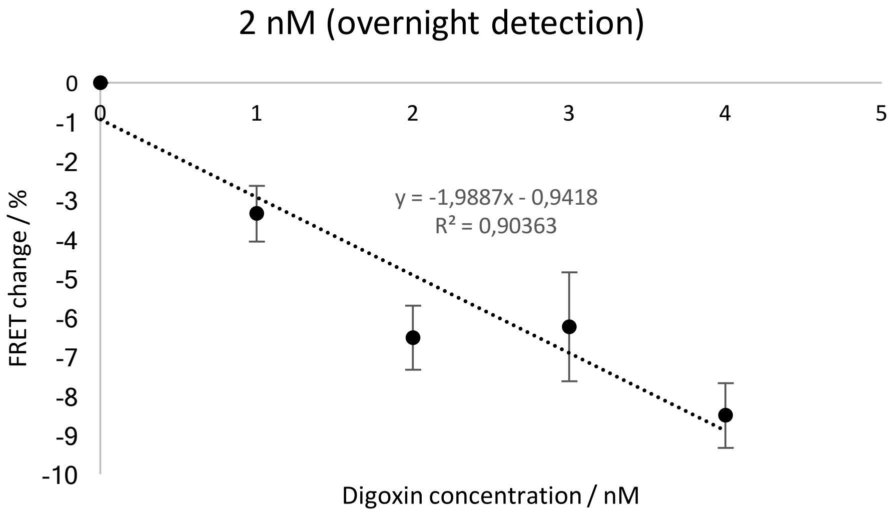

Figure S4: FRET ratio as a function of the digoxin concentration in the linear range of experiment from Figure 10 (0-4 nM of digoxin). LOD was calculated from the linear regression function (dashed line).

$$LOD\ (30\ min.detection) = \frac{3 * \sigma(10\ nM)}{\Delta FRET\ change} = \frac{3 * 0.442}{0.1614} = 8.2\ nM$$

$$LOD\ (overnight\ detection) = \frac{3 * \sigma(1\ nM)}{\Delta FRET\ change} = \frac{3 * 0.714}{1.9887} = 1.08\ nM$$
